# Regulation of Erk1/2 activation by osteopontin in PC3 human prostate cancer cells

**DOI:** 10.1186/1476-4598-9-260

**Published:** 2010-09-26

**Authors:** Brian W Robertson, Lauren Bonsal, Meenakshi A Chellaiah

**Affiliations:** 1 Department of Oncology and Diagnostic Sciences, Dental School, University of Maryland, Baltimore, MD21201 USA

## Abstract

**Background:**

Osteopontin (OPN) has been shown to play many roles in the progression of cancer. We have recently demonstrated the activation of Akt by OPN. Integrin-linked kinase and PI3-kinase are integral proteins in OPN/AKT pathway in PC3 cells. To investigate the role of the extracellular receptors in OPN signaling, we have examined the spatio-temporal regulation of CD44 and integrin αvβ3 receptor in OPN-induced Akt activation in PC3 cells.

**Results:**

Here, our studies demonstrate that OPN can activate Akt either through the α_V_β_3 _integrin or the CD44 cell surface receptor. Members of the Mitogen Activated Protein Kinase (MAPK) family have been shown to be up-regulated in a variety of human cancers and have been implicated in the metastatic behavior. Our studies have demonstrated an increase in the phosphorylation of c-Raf at Ser259 and Ser338 in PC3 cells over-expressing OPN. This increase matches up with the Erk1/2 phosphorylation at Thr202/204 and activation. However, the inhibition of Akt activity augments the phosphorylation state of ERK1/2 to two to three fold with a concomitant reduction in the phosphorylation state of c-Raf at Ser259.

**Conclusions:**

Regulation c-Raf phosphorylation at Ser259 has a role in the anti-apoptotic pathways mediated by Akt or Raf/MEK/ERK proteins. OPN may have dual effects in the activation of Erk1/2. We propose this based on the observations that while OPN activates c-Raf and Erk1/2; it also acts to inhibit c-Raf and Erk1/2 activation through Akt pathway. Our observations suggest that the activation of c-Raf-ERK cascade may promote cell cycle arrest in prostate cancer cells and OPN signaling has a role in the anti-apoptotic mechanism.

## Introduction

Osteopontin (OPN) is a multifunctional glycoprotein expressed by a number of cell types. Osteopontin expression has been linked to tumorigenesis and metastasis in a wide range of cancer types including prostate, breast, colon, melanoma, and lung [1]. Tumor bearing prostates contained 3.2 fold higher OPN levels [2]. OPN expression has been shown to be a prognostic indicator of survival among patients with advanced cancer. Elevated serum levels of OPN coincide with decreased survival rates among patients [3]. We have previously demonstrated that OPN has a role in osteoclast bone resorption [4] and prostate cancer cell migration [5], survival [6], and invasion [7].

Osteopontin mediates biological function through signal transduction by binding to the cell surface receptors such as integrin αvβ3 and CD44 [5]. It is an arginine-glycine-aspartic acid (RGD) containing extracellular matrix protein with diverse functions [8,9]. OPN interaction with integrin αvβ3 transduces cell-matrix signaling directed to increased motility, invasion, and angiogenesis [10]. Occupancy of RGD domain by αvβ3 elicits cell signaling required for cell migration and invasion [10,11]. Integrin αvβ3 and CD44 have a role in the metastasis of prostate cancer cells to bone by arbitrating adhesion to and migration on OPN protein present in the bone microenvironment [10-12].

The CD44 family of receptors regulates in a manner similar to that of integrins in cellular responses including adhesion, migration, and the stimulation of both cancerous and non-cancerous cells [13,14]. Our recent studies have shown an increase in the surface expression of CD44 isoforms (sCD44 and v4-v10 variant CD44) in prostate cancer cells over-expressing osteopontin (PC3/OPN) [15]. PC3 cells exhibited a rapid and strong adhesion to human bone marrow endothelial cell line (hBMECs), and depletion of CD44 expression by RNAi attenuated this adhesion [16]. Our most recent studies in prostate cancer cells demonstrate that OPN can activate Akt, an important step in cancer progression. An overall increase in β-catenin protein levels with a resultant transfer of β-catenin to the nucleus was observed in cells treated with or over-expressing OPN. Through the nuclear import of β-catenin, OPN increases both the transcription and protein levels of MMP-7 and CD44, which are known TCF/LEF transcription targets [6].

The Erk pathway is one of the best studied MAPK pathways in mammals and has been shown to be deregulated in approximately one-third of all human cancers [17]. Erk1/2 activation regulates proliferation, differentiation, survival, migration, angiogenesis, and even chromatin remodeling through the phosphorylation of both cytoplasmic and nuclear targets including phosphatases, transcriptional factors, and cytoskeletal proteins [17]. In the canonical Erk1/2 pathway, receptor tyrosine kinases are activated by specific ligands and trigger guanosine trisphosphate (GTP) loading of the Ras protein, which can then recruit the Raf kinases (A-Raf, B-Raf, c-Raf). These kinases consecutively phosphorylates and activates MEK (MEK 1 and MEK 2), ultimately leading to the activation of Erk1/2. In addition to this pathway, Erk1/2 has been shown to be activated by a variety of pathways depending on the individual ligand, cell surface receptor, and cell type [17]. Das et al. previously demonstrated that OPN induces AP-1 activation and uPA secretion through c-Src/EGFR/Erk signaling in breast cancer cells which ultimately control the motility in these cells [18]. Due to the existence of wide variation in the pathways leading to Erk1/2 activation, we investigated the OPN induced signaling pathway(s) which lead to Erk1/2 activation in prostate cancer cells and the role of cell surface receptors (αvβ3 and CD44) in this process.

Raf is targeted to the plasma membrane upon activation by a small GTPase. Phosphorylation of c-Raf at serine 259 is an inhibitory event occurring through Akt [19]. Previous studies have shown that osteoclast survival is dependent on the Erk1/2 signaling pathway [20]. Increased osteoclast production and activity contributes to excessive bone loss in conditions such as osteoporosis and tumor-induced osteolysis [20], which has been linked to prostate cancer [21]. Because prostate cancer results in metastases to the bone in approximately 80% of autopsied cases, prostate cancer cells present a logical system in which to study the relationships of bone extracellular matrix proteins and tumorigenesis [22]. OPN acts as a paracrine and autocrine mediator of prostate cancer growth and progression [11]. OPN role in the activation of MAPK pathway needs further elucidation. Therefore, we sought to determine how OPN promotes activation of the Erk pathway to induce cell proliferation. We have investigated the role of integrin αvβ3, CD44, and Akt by using SiRNA to CD44 and specific inhibitors to AKT and αv. We show here that elevated levels of OPN expression in prostate cancer cells stimulate Akt and Raf/MEK/ERK signaling pathways in order to produce different effects on proliferation and survival (anti-apoptosis).

## Results

### Osteopontin induces Erk1/2 activation

**Figure 1:**
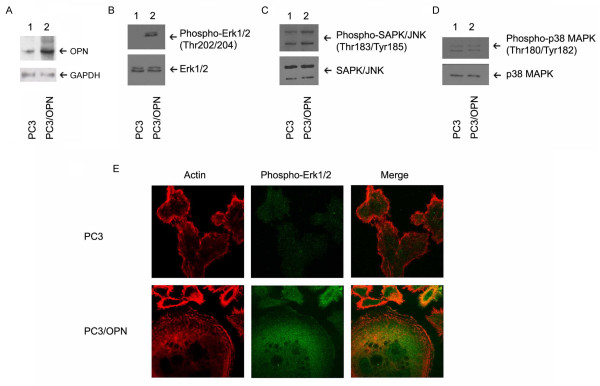
**OPN induces Erk1/2 activation**. Lysates from PC3 and PC3/OPN cell lines were immunoblotted with phospho-specific antibodies to OPN, Erk1/2, JNK, and p-38 (A-D, upper panels) proteins. Immunoblotting with the corresponding non-phosphorylated antibodies demonstrates equal loading of indicated proteins (A-D, lower panels). Confocal microscopy analysis of distribution of actin (red) and phospho-Erk1/2 (green) in PC3 and PC3/OPN cell lines is shown in Panel E. Results shown are representative of three independent experiments.

We measured the phosphorylation state of the three most widely known members of the mitogen-activated kinase (MAPK) family proteins including Erk1/2, JNK, or p38 MAPK in PC3 cells over expressing OPN (PC3/OPN). Stable PC3/OPN cells were generated as described previously [5]. PC3/OPN stable cell lines display an increased expression of OPN compared with stable PC3 cell lines expressing empty vector (Figure 1A, lane 2). Previous studies have shown that metastatic PC3 and DU145 prostate cancer cells have relatively low levels of active Erk1/2 [23]. Western blot analysis with indicated phosphor-specific antibody was performed. Consistent with those findings, we show here that PC3 cells expressing pCEP4 vector (henceforth represented as PC3) displayed either minimal or barely detectable levels of phosphorylation of Erk 1/2 (Figure 1B, lane 1). The phosphorylation is increased to a greater extent in PC3/OPN cells (Figure 1C, lane 2). An increase in the phosphorylation at Thr 202/204 represents the activation of Erk1/2 in PC3/OPN cells (Figure 1B, lane 2). Confocal analysis of PC3 and PC3/OPN cells stained for phospho-Erk1/2 also revealed a robust and diffuse staining of activated Erk1/2 in PC3/OPN cells (Figure 1E, bottom middle panel). An increased staining substantiates the activation of Erk1/2 in PC3/OPN cells since staining was performed with phosphor-Erk1/2 antibody. PC3 cells show sparse staining of phospho- Erk1/2 (Figure 1E, Top middle panel, green). This is consistent with the immunoblotting analysis shown in Figure 1B which demonstrates a decrease in the phosphorylation and activation of Erk1/2 in PC3 cells. Actin staining was used to demonstrate the cell periphery.

Immunoblotting analyses demonstrated a small increase in the phosphorylation of JNK at Threonine 183 and Tyrosine 185 in PC3/OPN cells (Figure 1C, lane 2). Furthermore, OPN had a very negligible effect on the phosphorylation of p38 MAPK at Thr180/Tyr182 (Figure 1D, lane 2). GAPDH was used as a loading control when probing total OPN expression levels (Figure 1A; lower panel). There were no observed differences in the protein levels of non-phosphorylated MAPK family members in either PC3 or PC3/OPN cell lines (Figure 1B-D; lower panels).

### Osteopontin induced Erk1/2 activation occurs through c-Raf and MEK1/2

**Figure 2:**
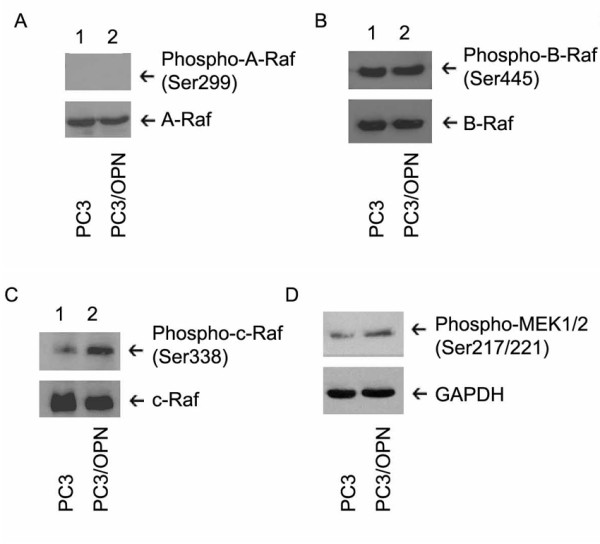
**OPN induces activation of c-Raf and MEK1/2**. Protein lysates from PC3 and PC3/OPN cell lines were immunoblotted with phospho-specific antibodies of A-Raf, B-Raf, c-Raf, and MEK1/2 (upper panels in A-D). Immunoblotting with the corresponding non-phosphorylated antibodies (lower panels in A-C) or GAPDH antibody (lower panel in D) was used to demonstrate equal loading. Results shown are representative of three independent experiments.

Raf and MEK have been shown to be the upstream regulators of Erk1/2 [17]. In order to determine the role of Raf and MEK1/2 in OPN-mediated activation of Erk1/2, western blot analysis was employed. Structures of the Raf proteins (A-Raf, B-Raf, and c-Raf) have been shown to be similar, but the proteins maintain differences in how they are activated and how they activate downstream targets such as MEK1/2 [17]. Activation of A-Raf and B-Raf is represented by the phosphorylation at Ser 299 and 245, respectively. Activation of c-Raf is measured by phosphorylation at Ser 338 [24]. Phosphorylation of A-Raf was almost not detected in PC3 and PC3/OPN cells (Figure 2A). Conversely, PC3 cells exhibited a higher basal level phosphorylation of B-Raf at Ser445 in PC3 cells (lane 1) and OPN expression had no effect in increasing the phosphorylation state of B-Raf (Figure 2B, lane 2). However, activation of c-Raf appears to highly dependent on OPN over-expression (Figure 2C, lane 2). An increase in the phosphorylation of c-Raf at Ser338 suggests that activation of c-Raf may have a role in the OPN-dependent Raf/MEK/ERK pathway and control apoptosis. Therefore we next proceed to investigate the activation of MEK1/2 in response to OPN over-expression. MEK1/2 activation is characterized by phosphorylation at two activation loop residues, Ser 217 and Ser 221. We found an increase in the activation of MEK1/2 in PC3/OPN cells as compared to PC3 control cells (Figure 2D).

### Akt negatively regulate Erk 1/2 activation in PC3/OPN cells

**Figure 3:**
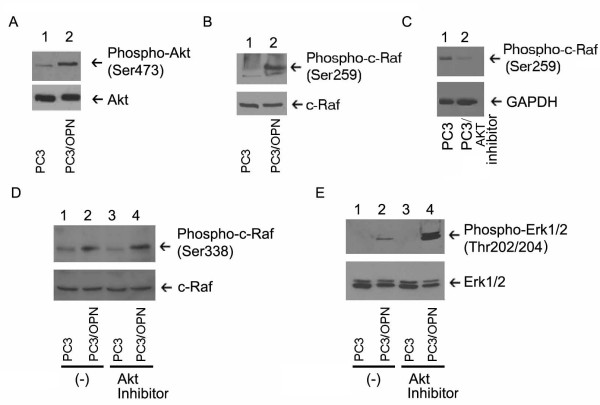
**Phosphorylation of OPN induced Erk1/2 and c-Raf activation by Akt**. (A) PC3 and PC3/OPN cells were immunoblotted with an Akt phospho-specific antibody (Akt-Ser473, upper panel). Membrane was stripped and blotted with an antibody to Akt to demonstrate total levels of AKT and equal loading (bottom panel). (B) PC3 and PC3/OPN cells were immunoblotted with an antibody to c-Raf Serine 259 (upper panel) and equal loading was shown using total levels of c-Raf (bottom panel). (C) PC3 cells were treated with either a vehicle or an Akt inhibitor overnight and immunoblotted with a phospho-specific antibody to c-Raf (Ser 259). (D and E) PC3 and PC3/OPN cell lines were pretreated with an Akt inhibitor for 16 h. The cell lysates were immunoblotted with phospho-specific antibodies to Erk1/2 (Thr202/204) or c-Raf (Ser338; upper panels in D and E). Membrane was stripped and immunoblotted with the corresponding non-phosphorylated antibodies to Erk1/2 and c-Raf to demonstrate equal protein loading (D and E-bottom panels). Results are representative of three independent experiments.

Recent observations have demonstrated an increase in the activation of Akt in PC3/OPN cells [6,25]. Little is known about the role of Akt in the Erk pathway in PC3 cells. Therefore, we have investigated the effects of Akt inhibitor on the phosphorylation of c-Raf and ERK1/2 on Thr202/204. OPN expression in PC3 cells increased Akt activation, as measured the phosphorylation of ser473 (Figure 3A, lane 2). Serine 259 of c-Raf has been shown to be regulated by Akt. Its phosphorylation provides a docking site for the cytosolic protein 14-3-3 and the subsequent inhibition of c-Raf activation [17]. OPN, presumably through Akt induces the phosphorylation of c-Raf at ser259 (Figure 3B). PC3 cells treated with Akt inhibitor showed an almost undetectable amount of c-Raf phosphorylation at ser259 (Figure 3C, lane 2) when compared with vehicle treated PC3 cells (Figure 3C, lane 1). In order to more fully understand the role of OPN in c-Raf activation and its association with Akt, the activation of Erk1/2 and c-Raf was studied in the presence of Akt inhibitor (Figure 3D and 3E). In the presence of an Akt inhibitor, PC3/OPN cells displayed a further increase in phosphorylation of c-Raf at Ser338 (lane 4 in Figure 1D) and Erk1/2 at Thr202/204 (Figure 3E, lane 4) as measured by immunoblotting analyses with respective phospho-specific antibody. These results indicate that while OPN ultimately activates c-Raf and Erk1/2, its activation of Akt plays an inhibitory role through the increased phosphorylation of c-Raf Serine 259, a known docking site for 14-3-3 protein.

### OPN induces activation of Akt through both α_V_β_3 _integrins and the CD44 cell surface receptor

**Figure 4:**
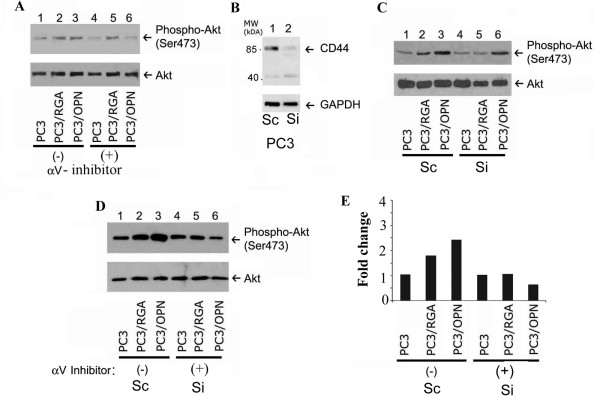
**Osteopontin induces activation of Akt through αVβ3 integrins and CD44**. Different PC3 cell lines (PC3, PC3/OPN, and PC3/RGA) were treated with and without α_V _integrin inhibitor (Cyclo-RGD, 10 μM for 16 h (panel A), siRNA to CD44 (200 nM for 48 hr; panels B and C), or a combination of both the α_V _integrin inhibitor and CD44 siRNA (panel D). The effects of inhibitors on Akt activation or phosphorylation were determined by immunoblotting analysis (upper panels, A-D). Total Akt levels were used as loading controls (lower panels in A, C, and D). Immunoblotting with a GAPDH antibody indicates equal loading in panel B. Changes in Akt phosphorylation levels were normalized to respective control on the Western blot shown in D and represented as fold change in the graph (Figure E). Graph E is a quantitative representation of the single western blot shown in panel D. Immunoblotting results are representative of three independent experiments.

Integrin αvβ3 and CD44 are receptors of osteopontin and CD44 is frequently over expressed in cancer cells [13]. To assess whether both the CD44 and αVβ3 receptors have a role in OPN- mediated Akt activation, we used a specific inhibitor (cyclo RGD; Figure 4A and 4D) to the αVβ3 integrin and siRNA to CD44 (Figure 4B and 4C). PC3 cells over expressing OPN with a mutation in the integrin binding domain RGDΔRGA (referred to as PC3/RGA) and thus no longer able to activate integrins were used to further define the individual roles of αVβ3 integrin and CD44 in the activation of Akt. The expression levels OPN and OPN (RGA) in these cell lines were shown previously. We do not see any differences in the molecular mass of cellular or secreted OPN in PC3, PC3/OPN or PC3/OPN (RGA) cells. The molecular mass of native OPN protein is approximately 30-36 kDa. These cells express ~60-68 kDa OPN protein which indicates that OPN is glycosylated [5]. PC3/OPN and PC3/RGA cells increase Akt activation (Figure 4A and 4C; lanes 2 and 3) when compared with PC3 cells, suggesting that OPN can induce activation of Akt in the absence of integrin signaling (Figure 4A and 4C, lane 1). In the presence of the αV inhibitor, PC3/OPN cells no longer have the ability to induce activation of Akt (Figure 4A, lane 6), while expression of mutant OPN in PC3 cells (PC3/RGA) did not affect the phosphorylation of Akt (Figure 4A, lane 5). The ability of PC3/RGA cells to activate Akt in the presence of the αV inhibitor suggests a role for an additional receptor.

CD44 is another receptor for OPN [13] and previous work from our laboratory showed that CD44 has an important role in the activation of MMP-9 and migration of PC3 cells [5]. Therefore, we sought to determine the role of CD44 in the activation of Akt using CD44 knock-down strategy with SiRNA to standard (s)CD44 (Figure 4B-E). We arrived at about 75-85% knockdown of sCD44 when using SiRNA to sCD44 (Figure 4B, lane 2). Scrambled RNAi was used as a control (lane 1). Mutation in OPN (RGA) abolishes Akt activation only in the cells depleted of CD44 (Figure 4C, lane 5) while PC3/OPN cells retain the ability to induce Akt activation, presumably through the interaction of αVβ3 and OPN via RGD-sequence (lane 6). However, cells treated with SiRNA to CD44 and an inhibitor to αv demonstrated a considerable decrease of both CD44 and αVβ3 integrin-mediated Akt activation (Figure 4E, lane 6). A graphical representation of changes in AKT phosphorylation (i.e. activation) is provided (Figure 4E) for the Western blot shown in Figure 4D. Cells treated with both αv-inhibitor and SiRNA to CD44 was normalized to the corresponding control cells untreated with αv-inhibitor but treated with scrambled RNAi (Figure 4E). These experiments illustrate that the interaction between OPN and either CD44 or integrin is sufficient to induce phosphorylation of Akt, which is largely responsible for the anti-apoptotic mechanisms vital to cancer cell survival and progression.

## Discussion

The ability of OPN to induce phosphorylation and activation of Erk1/2 represents a novel and important signaling mechanism in prostate cancer progression. Here we have identified that the increased expression of OPN leads to the activation of the Erk1/2 (Figure 1). Lack of OPN-mediated activation of JNK and p-38 proteins demonstrates that OPN does not stimulate the signaling pathways associated with these proteins. Signaling pathway analysis has revealed that Erk1/2 can be activated by a variety of upstream kinases and that each event is dependent on the specific ligand and cell type used [23,24]. The Raf/MEK/ERK cascade is known to be critically important in the regulation and growth of a variety of cells [26,27]. Previous studies have shown that inhibition of MEK1/2 resulted in the inhibition of Erk1/2 activation [17]. MEK1/2 was shown to be activated upon OPN over-expression and, due to the established role of MEK in Erk activation, we propose that this appears to be an important intermediary step in OPN-induced Erk1/2 activation (Figure 1). Of the Raf- family of proteins, increase in the phosphorylation of c-Raf at 338 represent an increase in the activation of this protein in the PC3/OPN cell line as compared with A-Raf and B-Raf. It seems that these proteins do not have a notable role in OPN mediated Erk1/2 signaling.

To further elucidate OPN signaling, we investigated the role of Akt in OPN mediated Erk1/2 activation. It has been shown that Akt plays an inhibitory role in both Erk1/2 and c-Raf activation through the phosphorylation of c-Raf at ser259, which facilitates the binding of 14-3-3 proteins [28]. We observed that the activation of Akt by OPN results in the phosphorylation of c-Raf259, which inhibits c-Raf activity and also decreases Erk1/2 activation (Figure 3). PC3/OPN cells treated with Akt inhibitor reveal an increase in the activation of Erk1/2 and c-Raf338 suggesting that Akt is acting as a negative regulator of Erk1/2 activation (Figure 3). Together, our results indicate that OPN has dual effects in the anti-apoptotic pathway. Osteopontin activates c-Raf and Erk1/2, while it also acts to inhibit c-Raf and Erk1/2 activation through Akt pathway.

**Figure 5:**
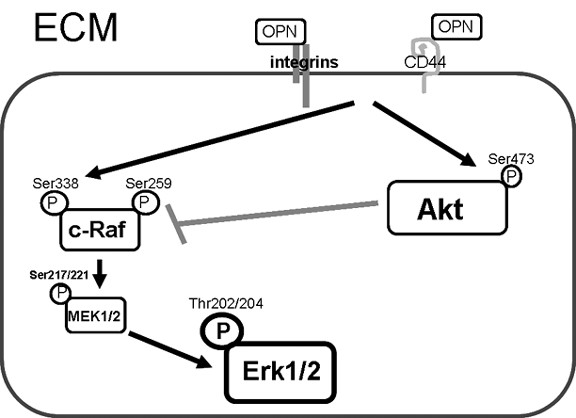
**Schematic model of signaling events of OPN induced Erk1/2 activation in prostate cancer cells**. OPN binds to PC3 cells by means of the CD44 receptor and integrins at the plasma membrane. Here we show that OPN phosphorylates c-Raf (Ser 338), a marker of c-Raf activation, leading to downstream activation of MEK1/2. OPN activates Akt as well. Akt activity has been shown to phosphorylate c-Raf (Serine 259) which is an inhibitory site of the Raf proteins therefore blocking increased activation of c-Raf. Thus, although OPN induces overall Erk1/2 activation, it also has an inhibitory effect on Erk1/2 through the activation of Akt.

Although high levels of active Akt are present in PC3 cells in the absence of OPN over-expression, we choose the PC3 cell line as a model system because they contain the cell surface receptors CD44 and αVβ3 integrins. We considered that this is the best model system to investigate the signaling interactions between OPN and each of these two surface receptors. The use of the cyclo-RGD molecular inhibitor of αvβ3 and SiRNA to CD44 in PC3 cell lines in combination with the use untreated (-) PC3 cell lines (over-expressing either full-length or mutated (RGDΔRGA) OPN) in figure 4 indicate that OPN can stimulate Akt activity through either αvβ3 or CD44 receptors (Figure 4). Upon mutation of the RGDΔRGA region, OPN still retains the ability to induce Akt activation presumably due to its interaction with CD44. Osteopontin is a ligand for several cell surface receptors, including αvβ3, αvβ1, α9β1, α4β1, α8β3, and CD44 [8,14]. To rule out the role of any additional surface receptors, we employed a combination of both CD44 siRNA and α_V_β_3 _integrin inhibitor and observed a loss Akt activation, indicating that binding of OPN to integrins other than αVβ3 does not result in a detectable level of Akt activation (Figure 4D). OPN binds to PC3 cells by means of the CD44 receptor and integrin αVβ3 at the plasma membrane in an arginine-glycine-aspartic acid (RGD) independent and dependent manner, respectively. A schematic diagram is provided as Figure 5 to demonstrate the role of OPN signaling in the anti-apoptotic mechanism.

Androgen-independent advanced prostate cancer cell lines such as DU145 and PC3 typically express low levels of activated Raf, MEK, and ERK [29]. In contrast to prostate cancer cells, breast cancer and hematopoietic cancer are typically associated with increased levels of Raf activation leading to increased proliferation and drug resistance. McCubrey et al. suggests that Raf/MEK/ERK may promote cell cycle arrest in prostate cancer cells and this may be regulated by p53 restoration [30,31]. Because introduction of wild type p53 into cell lines which have lost functional p53 such as PC3 and DU145 cell lines increases both the cells sensitivity to chemotherapeutic drugs and expression and activation of the Raf/MEK/ERK cascades [30,31]. Some have postulated that therapies aimed at increasing Raf activation may induce terminal differentiating senescence or cell cycle arrest in certain prostate cancers [32]. In advanced cancer it may be advantageous to induce Erk1/2 activation in order to promote cell cycle arrest, while in hematopoietic cancers it may be beneficial to inhibit Raf induced proliferation and drug resistance. Better understanding of how OPN works in tumorigenesis and in the MAPK signaling pathways may give insight into improved diagnosis, treatment, and prognosis of cancer.

## Methods

### Reagents

Monoclonal rabbit anti-phospho-p44/42MAPK (Erk1/2) (Thr202/Tyr204), anti-phospho-SAPK/JNK (Thr183/Tyr185), anti-phospho-c-Raf (Ser338), anti-p44/42MAPK (Erk1/2), anti-B-Raf, polyclonal rabbit anti-phospho-p38MAPK (Thr180/Tyr182), anti-phospho-c-Raf (Ser259), anti-phospho-c-Raf (Ser289/296/301), anti-phospho-A-Raf (Ser299), anti-phospho-B-Raf (Ser445), anti-p38MAPK, anti-SAPK/JNK, anti-A-Raf, and anti-c-Raf were purchased from Cell Signaling Technology (Danvers, MA). GAPDH and CD44 antibodies were purchased from Santa Cruz Biotechnology Inc (Santa Cruz, CA). OPN antibody was purchased from Rockland Immunochemicals (Gilbertsville, PA). Roswell Park Memorial Institute-1640 (RPMI-1640) media, fetal bovine serum (FBS), penicillin-streptomycin (10,000 units/ml penicillin and 10,000 μg/ml streptomycin), 0.25% Trypsin-EDTA, and phosphate buffered saline (PBS) pH 7.4 were purchased from Invitrogen (GIBCO; Auckland, NZ). Akt (5 μM) inhibitor, rhodamine phalloidin, and other chemicals were purchased from Sigma-Aldrich (St. Louis, MO). Protein assay reagent kit, reagents for polyacrylamide gel electrophoresis (PAGE), and molecular weight standards were purchased from Bio-Rad (Hercules, CA). Polyvinyldifluoride (PVDF) membrane for immunoblotting analysis was obtained from Millipore Corp. (Bedford, MA).

### Cell culture

Stable prostate cancer cell (PC3) lines that either over express unmutated OPN (PC3/OPN) or a mutant OPN in the integrin-binding site (PC3/RGDΔRGA) was generated as described previously [5]. PC3 cells transfected with empty pCEP4 vector were used as control. Cells were cultured at 37°C in RPMI-1640 media containing 10% Fetal Bovine Serum (FBS) and 1% Penicillin-Streptomycin. Upon reaching 100% confluency, cells were passaged with two brief phosphate buffered saline (PBS) washes, removed from tissue culture plates using 0.25% Trypsin-EDTA, and transferred to larger dishes.

### Preparation of cell lysates

Cells were washed two times with cold PBS and lysed in ice-cold RIPA lysis buffer (10 mM Tris-HCl, pH 7.2, 150 mM NaCl, 1% deoxycholate, 1% Triton X-100, 0.1% SDS, 1% aprotinin, 2 mM phenylmethysulfonyl fluoride, and 0.6 mM sodium orthovanadate). After incubating on ice for 10 min, lysates were centrifuged for 5 min at 6,000 rpm at 4°C. The supernatants were saved and protein concentrations were measured using the Bio-Rad protein assay reagent kit.

### Treatment of cells with inhibitors and immunoblotting with phospho-specific antibodies

PC3 cell lines (PC3, PC3/OPN, and PC3/OPN (RGA) were cultured in a 6-well culture dish and then treated with one of the following inhibitor in the presence of RPMI-1640 media containing 10% FBS at 37°C: Akt inhibitor for 16 h (1:500 dilution of 5 μM stock), αV integrin inhibitor (Cyclo-RGD, 10 μM for 16 h), siRNA to CD44 (200 nM for 48 h). CD44 siRNA (product number sc-29342) and scrambled siRNA (product number sc-37007) nucleotides were purchased from Santa Cruz Biotechnology Inc (Santa Cruz, CA). siRNA transfection reagent, RNAiFect, was purchased from Qiagen (Valencia, CA). Protein lysates were subjected to 12% SDS-PAGE and Western blot analysis as described below.

### Western blot analysis

Cell lysates were denatured by boiling for 5 minutes in Novagen 1× SDS sample buffer (EMD Biosciences, Inc. Madison, WI). Proteins were resolved by SDS-polyacrylamide gel electrophoresis on 8% or 12% gels and then transferred to PVDF membranes. The membranes were initially blocked with PBS containing 0.05% TWEEN 20 (PBS-T) and 5% BSA for 1 h at room temperature (RT) and were then probed overnight at 4°C using a dilution of 1:1000 with the following primary antibodies in PBS-T and 5% BSA: anti-phospho-p44/42MAPK (Erk1/2) (Thr202/Tyr204), anti-phospho-SAPK/JNK (Thr183/Tyr185), anti-phospho-c-Raf (Ser338), anti-phospho-p38MAPK (Thr180/Tyr182), anti-phospho-c-Raf (Ser259), anti-phospho-c-Raf (Ser289/296/301), anti-phospho-A-Raf (Ser299), and anti-phospho-b-Raf (Ser445). After three washes with PBS-T for 5 minutes each, the membranes were incubated with a 1:1000 dilution of species-specific horseradish peroxidase (HRP)- linked secondary antibody (Little Chalfont, UK) in PBS-T and 5% blotting grade blocker non-fat dry milk (Bio-Rad, Hercules, CA) for 2 h at RT. Blots were washed three times with PBS-T for 15 min. each. Protein bands were visualized by chemiluminescence using a SuperSignal West Pico Chemiluminescent Substrate Kit (Thermoscientific, Rockford, IL). PVDF membranes were stored in PBS-T at 4°C until being stripped and re-probed with the corresponding control antibodies to determine the loading in each lane as described below.

### Stripping and reprobing of membrane with antibody of interest

The PVDF membranes were incubated in stripping buffer (2% sodium dodecyl sulfate (SDS), 62.5 mM Tris HCl pH 7.2, and 100 mM β-mercaptoethanol) at 55°C for 15 min. After three washes with PBS-T for 15 minutes each, the membranes were blocked with PBS-T and 5% blotting grade blocker non-fat dry milk (Bio-Rad, Hercules, CA) for 1 h at room temperature (RT) and were then probed overnight at 4°C using a dilution of 1:1000 of the primary antibody of interest (e.g. anti-GAPDH at 1:1000 dilution) in PBS-T and 5% blotting grade blocker non-fat dry milk. The membranes were washed three times with PBS-T for 5 min each and were then incubated with a 1:1000 dilution of species-specific horse-radish peroxidase (HRP-) linked secondary antibody in PBS-T and 5% blotting grade blocker non-fat dry milk (Bio-Rad, Hercules, CA) for 3 h at RT. Membranes were washed and proteins bands were visualized as described above.

### Immunostaining analysis

PC3 and PC3/OPN cells were cultured onto cover slips in a 12-well dish for 14-16 h at 37°C. Cells were washed three times with room temperature PBS (RT-PBS) and fixed in 4% formaldehyde-PBS for 10 min. After washing three times with RT-PBS, cells were permeabilized with 0.5% Triton X-PBS for 10 min. Cells were washed three times with RT-PBS, followed by incubation in 5% boiled goat serum for 1 h at RT. After washing three times with RT-PBS, cells were incubated with a 1:100 dilution of anti-phospho-p-44/42(ERK1/2) (Thr202/Tyr204) in 5% boiled goat serum overnight at 4°C. Cells were washed three times with RT-PBS. Subsequently, cells were incubated for 3 h at RT in the dark with the following: 1:1000 dilution of FITC-conjugated species specific secondary antibody and 1:500 dilution of rhodamine phalloidin for actin staining. Cells were washed three times with RT-PBS for 15 minutes each and the cover slips were transferred cell side down onto glass slides containing perma fluor mounting medium (Thermo Scientific, Pittsburgh, PA) and sealed with clear nail polish around the edge of the cover slips. The immunostained cells were viewed and photomicrographed on a Bio-Rad 6000 (Hercules, CA) confocal microscope. Images were stored in TIF image format and processed by the Adobe Photoshop software program (Adobe Systems, Inc., Mountain View, CA).

## List of abbreviations

OPN: Osteopontin; RGD: arginine-glycine-aspartic acid sequence. It is known as integrin binding motif; αvβ3: vitronectin receptor; sCD44: soluble or standard CD44; PC3/OPN: PC3 cells over expressing osteopontin; PC3/RGA; PC3 cells expressing mutated OPN in RGD Δ RGA motif where aspartic acid (D) Δ Alanine (A); ERK: extracellular signal regulated kinase; MAPK: mitogen activated protein kinase; FBS: fetal bovine serum; GAPDH: glyceraldehydes-3-phosphate dehydrogenase; FITC: fluorescein isothiocyanate

## Competing interests

The authors declare that they have no competing interests.

## Authors' contributions

BWR and MAC participated in the design of the study and wrote the manuscript. BWR and LB carried out the experiments. All authors read and approved the final manuscript.
